# GENESISS 2—Generating Standards for In-Situ Simulation project: a systematic mapping review

**DOI:** 10.1186/s12909-022-03401-y

**Published:** 2022-07-11

**Authors:** Kerry Evans, Jenny Woodruff, Alison Cowley, Louise Bramley, Giulia Miles, Alastair Ross, Joanne Cooper, Bryn Baxendale

**Affiliations:** 1 Nottingham University Hospitals Trust, Institute of Care Excellence, Nottingham, UK; 2 Birmingham Children’s Hospital, Birmingham, UK; 3 Nottingham University Hospitals Trust, Research & Innovation, Nottingham, UK; 4 Trent Simulation & Clinical Skills Centre, Nottingham University Hospitals NHS Trust, Nottingham, Notts UK; 5 Glasgow Dental School, University of Glasgow, Glasgow, UK

**Keywords:** In-situ simulation; simulation-based education, Clinical training, Simulated practice, Health professions

## Abstract

**Background:**

In-situ simulation is increasingly employed in healthcare settings to support learning and improve patient, staff and organisational outcomes. It can help participants to problem solve within real, dynamic and familiar clinical settings, develop effective multidisciplinary team working and facilitates learning into practice. There is nevertheless a reported lack of a standardised and cohesive approach across healthcare organisations. The aim of this systematic mapping review was to explore and map the current evidence base for in-situ interventions, identify gaps in the literature and inform future research and evaluation questions.

**Methods:**

A systematic mapping review of published in-situ simulation literature was conducted. Searches were conducted on MEDLINE, EMBASE, AMED, PsycINFO, CINAHL, MIDIRS and ProQuest databases to identify all relevant literature from inception to October 2020. Relevant papers were retrieved, reviewed and extracted data were organised into broad themes.

**Results:**

Sixty-nine papers were included in the mapping review. In-situ simulation is used 1) as an assessment tool; 2) to assess and promote system readiness and safety cultures; 3) to improve clinical skills and patient outcomes; 4) to improve non-technical skills (NTS), knowledge and confidence. Most studies included were observational and assessed individual, team or departmental performance against clinical standards. There was considerable variation in assessment methods, length of study and the frequency of interventions.

**Conclusions:**

This mapping highlights various in-situ simulation approaches designed to address a range of objectives in healthcare settings; most studies report in-situ simulation to be feasible and beneficial in addressing various learning and improvement objectives. There is a lack of consensus for implementing and evaluating in-situ simulation and further studies are required to identify potential benefits and impacts on patient outcomes. In-situ simulation studies need to include detailed demographic and contextual data to consider transferability across care settings and teams and to assess possible confounding factors. Valid and reliable data collection tools should be developed to capture the complexity of team and individual performance in real settings. Research should focus on identifying the optimal frequency and length of in-situ simulations to improve outcomes and maximize participant experience.

## Background

In-situ simulation (ISS) training enables teams to practice and be assessed in their own, familiar clinical environments [1, 2]. ISS is often focused on training for low volume, high impact emergencies involving multidisciplinary teams (MDTs) with the aim of reinforcing knowledge and improving the functioning of the clinical team as a whole [3–5]. The main benefit of ISS over other traditional simulation approaches is reported as allowing participants to problem solve within their own dynamic setting which supports the implementation of learning into practice [1, 2].

ISS has been identified as a useful mechanism to explore and learn from adverse events [6–9]. Embedding ISS activities underpinned by Human Factors principles can help to focus on the organisational, procedural and contextual influences on clinical reasoning and actions [10, 11]. ISS has also been developed to test the synergy or dissonance between micro and macro factors: task factors, organisational factors, internal environments and external environments [12]. ISS interventions have been reported as a mechanism to enhance patient flow, improve the design of clinical spaces, and identify latent safety threats (LSTs) within new clinical settings [13–16]. The ability to experiment and see what occurs through interactions, attunement and disturbances enables participants to try out different options and consider possible unintended outcomes [17].

Organisational resilience is focused on understanding how healthcare organisations can deliver standardised, replicable and predictable services while embracing inherent variations, disruptions and unexpected events [18]. During the Covid-19 pandemic, ISS proved useful in helping teams prepare in a rapidly emerging situation. ISS interventions included testing and implementing the use of personal protective equipment (PPE), infection control guidelines and supporting operational readiness of intensive care units and operating rooms [19–23]. ISS interventions are employed to improve the acquisition of NTS, task management, situation awareness, problem-solving, decision-making and enhancing teamwork while testing and probing real-world organisational systems [1, 18, 24–27].

ISS offers a feasible and acceptable approach through which individual and team competency can be assessed through simulated scenarios in controlled and standardised clinical settings [28]. Griswold et al. [29] identify that summative assessment using ISS is suited to clinical procedures with clear chains of action and well-defined processes and standards. Clinical competency measurement and assessment tools are less well-defined for ISS and further complicated when individual performance needs to be isolated from the wider team. Concepts such as ‘effective communication’ are subject to interpretation, and clinical outcomes may be attributed to concepts such as teamwork and coordination in addition to individual clinical skills and knowledge [30].

**Fig. 1:**
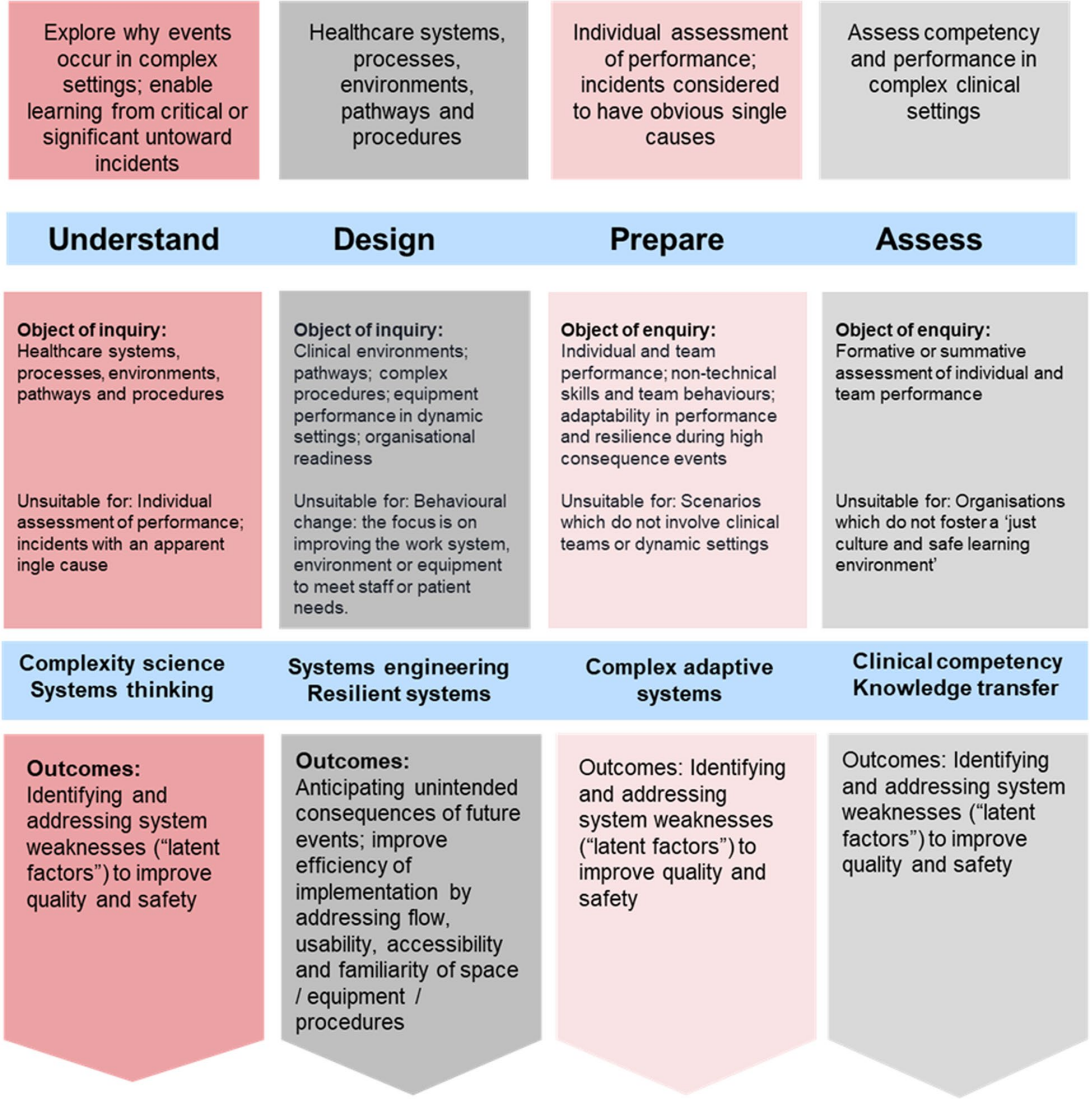
Conceptual Model of In-Situ Simulation in Healthcare

Although ISS has been identified as a promising approach in healthcare settings, ISS terms and concepts require standardisation and integrated models of learning are required to provide a more comprehensive and cohesive strategic approach [1, 31, 32]. The overall aim of the Generating Standards for In-Situ Simulation project phase 2 (GENESISS -2) was to develop evidence-based standards for healthcare professionals, educators and managers interested in developing and implementing ISS interventions in clinical practice. The project was commissioned by Health Education England working across the Midlands and East. A conceptual model of ISS was developed in phase one [33] which proposed four main ISS functions (Fig. 1). The aim of this systematic mapping review was to: explore and map the current evidence base for ISS approaches, identify gaps in the literature and inform future research questions.

## Methods

We chose to conduct a systematic mapping review to capture the wide evidence base on main uses of ISS in healthcare. Mapping reviews are specifically designed to describe the extent of research in a field, spanning broad topic areas and research objectives to identifying evidence gaps to be addressed by future research [34]. The report follows the PRISMA (Preferred Reporting Items for Systematic Reviews and Meta-Analyses) statement guidelines [35]. The review protocol was registered on the PROSPERO database (CRD42019128071). Recommendations for systematic mapping reviews [36–38] guided the review conduct.

### Search

The search strategy was developed for MEDLINE, EMBASE, AMED, PsycINFO, CINAHL, MIDIRS and ProQuest databases and completed the literature search in March 2019 and updated in October 2020. A summary of the search terms is included in Table 1 and supplementary file 1 provides details of the full Medline search strategy.

**Table 1:** Search terms

Simulation training / teaching Simulation education Patient simulation Simulation High / Low fidelity simulation Experiential learning Drill Mannequin	and	In-situ In practice Work based Workplace Point of care Real world Mobile Cart Hospital / ward Primary care Clinic On-site Patient area	and	Patient safety Human factor Adverse event Harm / risk / incident Clinical governance Outcome assessment Patient reported outcomes Quality improvement Medical errors Clinical competence / skill Technical skill Non-technical skill Interpersonal skill Situational awareness Performance Capability / expertise Communication Knowledge (transition / translation) Leadership Handover / off Organisational (departmental) efficiency / performance Pathway / care / flow Cost benefit Economic / cost Orientation

Papers were included in the review if they met the following criteria: (i) published in English, (ii) based in an Organisation for Economic Co-operation and Development (OECD) member country (to enable greater comparability between health systems and socio-economic contexts), (iii) reporting quantitative primary research including randomised controlled trials, quasi-experimental studies, cohort studies, economic evaluation and observational quantitative studies (iv), included healthcare practitioners as participants (individual and teams) (v) reported simulation training or interventions conducted in any patient care settings (vi) reported quantitative measures of safety, governance, quality improvement, technical and non-technical skills performance, and educational or clinical outcomes. Exclusion criteria were (i) papers reporting simulation activities conducted in educational institutions and centres, simulation laboratories or training suites or non-patient areas (ii) qualitative studies, secondary data analysis and literature reviews. The timeframe for inclusion was from inception to October 2020.

Papers retrieved from the literature databases were imported to an EndNote library, and duplicate records were identified. Two researchers independently screened the titles and abstracts against the review inclusion and exclusion criteria (KE, JW). Full text papers of the remaining citations were then retrieved and independently assessed by two researchers (first stage: KE, JW updated search: KE, AC). A third researcher (BB) moderated any discrepancies until the final selection of papers was agreed upon.

### Quality assessment

The quality of studies included in the review was evaluated using a range of established critical appraisal tools selected for the particular study design: Quality Assessment Tool for Before-After (Pre-Post) Studies with No Control Group [39]; The Cochrane Risk of Bias tool for Randomised Controlled Trials [40]; The Joanna Briggs Institute (JBI) Checklist for Quasi-Experimental Studies [41]; CASP tool for cohort studies [42]. Two independent researchers assessed study quality (first stage: KE, JW updated search: KE AC) and banded studies as low, medium and high quality. There was consensus between the two researchers. Although no studies were excluded on the basis of quality, the quality assessment was used to identify the strengths and limitations of the review [43]. JBI levels of evidence [44] for included studies was also reported.

### Data extraction

Data extraction forms were designed and piloted before beginning data extraction, completed by two independent researchers. Data extraction tables consisting of numerical and textual data presented the study characteristics, results and quality assessments.

### Data analysis and synthesis

Synthesis of the extracted data were conducted in a descriptive and tabular way [45]. Categories were developed through an iterative process, focusing on the main aims or purposes of ISS interventions, illustrating the range of methods, intervention components, duration, populations, outcome measures and gaps in the research within and between each category. A description of the quantitative data is presented in tables to enhance explanation, understanding and coherence of the findings [37].

## Results

**Fig. 2:**
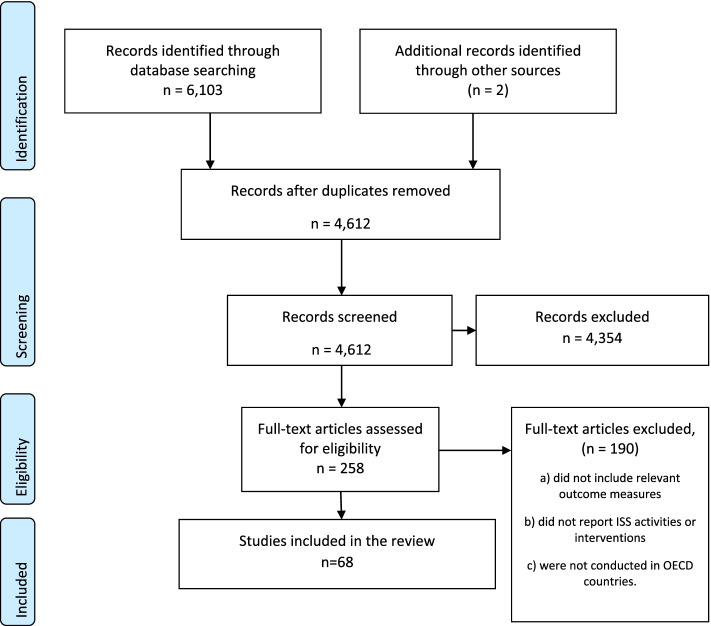
PRISMA diagram: In-situ simulation to improve safety, effectiveness and quality of care

The search identified 6,105 potentially eligible papers. Duplicate papers were removed (*n* = 1493). Papers were then screened (4,612) based on the information provided by the title and abstract. Potentially eligible papers (*n* = 258) were retrieved for full text assessment by two independent reviewers (KE, JW) and any disagreement resolved by discussion with a third reviewer (BB) until agreement was reached. The level of agreement between the two reviewers produced a kappa value of 0.9 which suggests a very good strength of agreement (k = 0.9, *p* < 0.001). Excluded papers (*n* = 189) a) did not include relevant outcome measures, b) did not report ISS activities or interventions c) were not conducted in OECD countries. The literature search and inclusion process are detailed in the PRISMA Flow diagram [46] (Fig. 2). There were 68 papers included in the mapping review which met the inclusion criteria.

Findings were organised into categories to reflect the aims and objectives of the included studies using ISS: 1) as an assessment tool; 2) to assess and promote system readiness and safety cultures; 3) to improve clinical skills and patient outcomes; 4) to improve NTS, knowledge and comfort and confidence. The themes presented are:

### ISS to assess performance and identify risks

Eighteen studies conducted ISS as a method of assessment (Table 2). Studies were conducted in the US, Canada, Denmark, Sweden, UK, Germany, Switzerland. Most studies were observational (*n* = 17), with one study reporting a quasi-experimental design to compare outcomes using different resuscitation equipment [47]. Samples sizes (where reported) ranged from 12 to 277 participants. Five studies reported ISS interventions to assess performance and identify risks: medication errors in emergency departments [48], LSTs in a Children’s medical centre [49], paediatric and neonatology departments [50], pediatric tracheostomy care management in Emergency Departments (EDs), Intensive Care Units (ICUs) [51], and blood transfusion policies in the operating room [52]. Four studies reported ISS interventions to assess compliance against clinical guidelines and standards: cardiac arrest guidelines [53], sepsis guidelines [54], blood transfusion policy and identification [52] and cardiopulmonary resuscitation (CPR) performance [55]. Four studies reported ISS interventions to assess clinical response and task completion time [56–59], with three studies employing a pre / post ISS evaluation to evaluate the effectiveness of training programmes [60–62]. ISS was used to test and assess the safety of new equipment and procedures in two studies: the use electronic health records in the ICU [63] and to assess and compare traditional and automated external defibrillator supplemented responder models [47]. One study [64] conducted ISS to assess performance-relevant effects of task distribution and communication amongst emergency teams.

**Table 2:** ISS to assess performance and identify risks

**Author, date** (Country)	**Research topic**	**Setting and participants**	**Outcome methods and measures**	**Study type (JBI level of evidence)**
**Auerbach 2018 **[45] (US)	Adherence to paediatric cardiac arrest guidelines	ED (50 ED departments), MDT teams	1. Cardiac arrest adherence score (AHA guidelines) 2. Timing and task completion 3. Simulated Team Assessment Tool (STAT)	Prospective observational *Quality assessment – good* (3e)
**Calhoun 2014 **[46]** (US)**	Task performance in ICU examining work length on task completion	Paediatric ICU, nurses (*n* = 28)	1. Task completion via direct observation	Prospective observational *Quality assessment: low – moderate* (3e)
**Campbell 2016 **[47] (Canada)	Blood administration processes and hazards	Operating room, HCPs (*n* = 43)	1, Adherence to a process checklist 2. Identification of latent hazards 3. Performance and teamwork ANTS tool and CTS	Prospective observational *Quality assessment—good* (3e)
**Clapper 2018 **[48] (US)	Assess the impact of the saturation-in-training model of TeamSTEPPS implementation	Paediatric in-patient unit (*n* = 547, ISS with smaller sub-sample)	1. Participant TeamSTEPPS knowledge scores 2. TeamSTEPPS performance scores	Prospective observational *Quality assessment – good* (3e)
**Hargestam 2015 **[49] (Sweden)	Time taken to make a decision to go to surgery	ED, trauma teams (*n* = 96 participants)	1. Clinical management timings 2. Communication 3. Leadership style	Prospective observational *Quality assessment: moderate* (3e)
**Kessler 2016 **[50] (US)	Adherence to paediatric sepsis guidelines	Paediatric ED, MDT teams (*n* = 47 teams)	1. Compliance with International sepsis guidelines 2. Experience and attitudes to sepsis care	Prospective observational *Quality assessment: good* (3e)
**Kobayashi 2010 **[51] (US)	Comparing defibrillators in the ED	Hospital, nurses (*n* = 50)	1. Resuscitation performance	Quasi-experimental *Quality assessment: moderate* (2d)
**Kozer 2004 **[52] (Canada)	Characterise the incidence of medication errors	Paediatric ED, 20 physicians, 15 nurses	1. Drug type and drug concentration administered	Prospective observational (3e)
**Lipman 2013 **[53] (US)	Assess performance of response times for emergency caesarean delivery	Maternity unit, MDT (*n* = 14 teams)	1. Timings to perform emergency caesarean 2. Barriers to optimal team performance	Prospective observational *Quality assessment: moderate* (3e)
**Lipman 2013b **[54] (US)	Assess performance of CPR during obstetric crisis in different settings	Maternity unit, MDT (*n* = 14 teams)	1. Correctly delivered chest compressions 2. CPR skills	Prospective observational *Quality assessment: moderate* (3e)
**Lok 2014 **[55] (UK)	Identifying latent risks in paediatrics and neonatology	Paediatrics and neonatology MDT (*n* = 10 hospitals, *n* = 246)	1. Latent risks (NPSA recommendations)	Prospective observational *Quality assessment: moderate* (3e)
**March 2013 **[56] (UK)	Establish the role of simulation training to test the efficacy and safety of the electronic health record	ICU Medical staff (*n* = 38)	1. identification of action items and clinical trends (patient condition / medical error)	Prospective observational (3e)
**Mondrup 2011 **[57] (Denmark)	CPR performance	Hospital staff (first responders)	1. CPR performance using the Laerdal PC Skill Reporting System based on established ALS guidelines	Prospective observational *Quality assessment: moderate* (3e)
**Author, date (Country)**	**Research topic**	**Setting and participants**	**Outcome methods and measures**	**Study type (JBI level of evidence)**
**Sarfati 2015 **[58] (France)	Prevent medication errors	Hospital Pharmacy technicians (*n* = 12)	1. Detection of medication errors pre and post awareness training	Prospective observational *Quality assessment: moderate – good* (3e)
**Shah 2020 **[59] (US)	Identify LSTs assess care safety	Paediatric and neonatal ICU and ED (*n* = 65, 21 simulations)	1.Assess the clinical environment and identify LSTs 2.Analyse effects of educational and systems interventions 3.Determine which team factors and interventions were associated with better simulation performance	Prospective observational *Quality assessment: moderate – good* (3e)
**Schmutz 2015 **[60] (Germany)	Task distribution and communication in emergency teams	Hospital emergency teams paediatric wards (*n* = 277)	1. Team behaviour 2. Clinical performance using published clinical checklists	Prospective observational *Quality assessment: moderate* (3e)
**Wheeler 2013 **[61] (US)	Improve quality of care delivered to children with impending respiratory or cardiopulmonary arrest	PICU, CICU, OR, patient care units	1. Identification of latent safety threats 2. Participant evaluation	Prospective observational *Quality assessment: moderate* (3e)
**Zimmermann 2015 **[62] (Switzerland)	Evaluate resuscitation training	Children’s hospital, MDT (*n* = 95)	1. Team performance 2. LST identification	Prospective observational *Quality assessment: good* (3e)

*ED* Emergency Department, *ICU* Intensive Care Unit, *MDT* Multi Disciplinary Team, *PICU* Paediatric Intensive Care Unit, *OR* Operating Room, *CICU* Cardiac Intensive Care Unit

Auerbach et al. [45] and Kessler et al. [50] employed voluntary participation for ISS assessments, although the authors discussed that selection bias may be introduced as individuals agreeing to participate may be more or less skilled than other staff [53]. In addition scheduling of ISS may have resulted in providers and departments preparing for the day (training effect). Lipman et al. [53] reported that clinical timings may have been underestimated due to participation of highly skilled teams, the close proximity of clinical departments and participants to the drill area, absence of patient family members, participant knowledge of the imminent ISS activity and training conducted during daytime hours [55, 58]. Involvement of participants without other clinical duties at a scheduled announced time may limit the generalisability of the findings [53].

ISS performance was assessed by direct observation and by accessing feedback from participants. Two studies used evidence based clinical standards to assess performance, quality and safety metrics [53, 54]. Outcome measures based on established standards were reported to be easily measurable, reproducible, and reflect clinical metrics and benchmarks. However, ISS assessment can be limited by the inability to reliably assess the impact on clinical outcomes due to the low occurrence of critical events [61], and poor sensitivity of outcome measures to assess communication skills in functional teams [57]. Most of the included studies used locally developed checklists, developed through previous pilot testing or amended from checklists developed for other clinical settings. Studies which reported team and system level assessments used established outcome measures including the Simulation Team Assessment Tool [53, 65], Anaesthetists' non-technical skills (ANTS) taxonomy and behaviour rating tool [66, 67], TeamSTEPPS Team Performance Observation Tool [60, 68].

Authors reported positive benefits of conducting ISS to identify risks and hazards in clinical environments and improve the ability to detect errors. ISS was reported to help identify system susceptibilities, evaluate the effectiveness of training programmes and highlight variability in performance across different departments and systems. Overall, aauthors reported positive benefits of ISS as a method of assessment, providing useful information to inform future improvement initiatives.

### ISS to assess and promote system readiness and safety cultures

Nine studies conducted ISS interventions with the aim of improving system or departmental performance outcomes (Table 3). Studies were conducted in Denmark, the UK and US. All studies were observational, and data were collected via participant questionnaires, and/or direct observation (or a review of audio-visual recordings) by trained assessors or experienced clinicians. Five studies were conducted in EDs [69–73], two in operating theatres [74, 75], one in a neonatal ICU [13] and one in an obstetric unit [76]. Samples sizes (where reported) ranged from 14 to 289 participants. ISS interventions varied from single training sessions to regular training sessions over a period of months. All studies included participants from multi professional healthcare teams. Studies reported ISS was used as a way to assess, prepare and orient staff to new facilities [70–72, 76, 77] and promote safety cultures across departments or systems [69, 73–75]. All of the studies reported improvements in readiness scores and safety attitudes outcomes.

**Table 3:** ISS to assess and promote system readiness and safety cultures

Author, date (Country)	Research topic	Setting and participants	Outcome methods and measures	Study type (JBI level of evidence)
**Abulebda 2018 **[70]** (US)**	Assessing paediatric readiness and adherence to guidelines	ED (10 ED departments), MDT teams (*n* = 41)	1. Paediatric Readiness Score	Prospective observational *Quality assessment – moderate* (3e)
**Bender 2011 **[13]** (US)**	Improve system readiness and staff preparedness in a new NICU	Neonatal ICU (*n* = 148)	1. System readiness TESTPILOT 2. Identification of LSTs 3. Staff preparedness	Prospective observational *Quality assessment: moderate* (3e)
**Gardner 2013 **[71]** (Canada)**	ED preparedness: LST detection, orientation, preparedness	ED (*n* = 55)	1, System readiness 2. Workplace satisfaction	Prospective observational *Quality assessment—good* (3e)
**Hinde 2016 **[74]** (UK)**	Improve safety culture of operating theatres	OR (*n* = 72)	1. Safety attitude questionnaire 2. Safety Climate scores 3. Teamwork scores	Prospective observational *Quality assessment – moderate* (3e)
**Jaffry 2019 **[75]** (UK)**	Enhance compliance with safety checklists and promote the safety culture	(*n* = 25)	1. Knowledge and confidence scores 2. Compliance with the WHO Surgical Safety Checklist	Prospective observational *Quality assessment: moderate* (3e)
**Kobayashi 2006 **[72]** (US)**	Evaluate the capacity of a new ED for emergent resuscitative processes and assist facility orientation	ED (*n* = 14)	1. Staff preparedness 2. Orientation scores	Prospective observational *Quality assessment – moderate* (3e)
**Paltved 2017 **[73]** (Denmark)**	Enhance patient safety attitudes	(*n* = 39)	1. Safety attitude questionnaires 2. Safety climate scores 3. Teamwork scores	Prospective observational *Quality assessment: good* (3e)
**Patterson 2013 **[69]** (US)**	To decrease the frequency and mitigate the effects of medical error	Paediatric ED (*n* = 289 / *n* = 151)	1. Safety climate scores 2. Teamwork climate scores	Prospective observational *Quality assessment: good* (3e)
**Ventre 2014 **[76]** (US)**	Evaluate operational readiness	Children’s hospital obstetric unit (*n* = 133)	1. LST detection rate 2. Equipment checklists	Prospective observational *Quality assessment: moderate—good* (3e)

*ED* Emergency Department, *ICU* Intensive Care Unit, *MDT* Multi Disciplinary Team, *PICU* Paediatric Intensive Care Unit, *OR* Operating Room, *CICU* Cardiac Intensive Care Unit

Data were mainly collected via pre and post participant self-assessment questionnaires, outcomes included identification of LSTs, assessment of departmental readiness scores, safety cultures and attitudes, orientation and team and departmental performance. Identification of LSTs was captured via observation and via participant during ISS debriefing.

Ventre et al. [76] identified that although clinicians participated in a basic orientation to the new space, ISS provided additional opportunity to evaluate whether the electronic and information systems, equipment and devices performed adequately before opening. Kobayashi et al. [72] conducted ISS when a new ED was almost ready to open, yet with enough time remaining for adjustments and corrective actions on identified issues. However, ISS may assist not only in testing the new facility but also in designing the environments [78].

Three studies conducted ISS to improve safety compliance, cultures and attitudes [73–75]. Although safety and teamwork climates were reported as readily measured and amenable to improvement through ISS, it was difficult to demonstrate an association between team and safety training on patient outcomes as improved clinical outcomes are multifactorial [74], evaluating the role of team versus organisational processes can be challenging [73]. Paltved et al. [73] discussed how prolonged engagement with ISS interventions and longer follow-up periods may be required as safety attitudes do not suddenly appear but emerge over time. Jaffrey et al. [75] reported that ISS emphasises the importance of safety measures and empowers participants to make changes and implement them effectively. ISS provides both a learning and a working environment which incorporates the complexity and resources found in the clinical environment and supports knowledge transfer to actual practice [73].

### ISS to improve clinical skills, performance and clinical management

Seventeen studies conducted ISS interventions with the aim of improving clinical skills, performance and clinical management (Table 4). Studies were conducted in Australia, Israel, Italy, the UK and US. Ten studies were Pre / Post observational studies which included ISS interventions, two were prospective cohort studies, two RCTs, one observational study with a control and one multicomponent quality improvement project. Studies were conducted in emergency and resuscitation teams and departments [79–86], paediatric and neonatal care settings [87–89], in-patient ward settings [90–92], coronary care [93], an obstetric unit [94] and a mental healthcare setting [2]. Where reported, ISS interventions frequency varied from single training sessions delivered over one day to repeat ISS training lasting 18 months. The length of ISS was reported to last 30 min to 3 h. Most studies included participants as multi professional healthcare teams, with two studies including doctors and one including only nurses. Sample sizes ranged from 22–303 participants. ISS frequency, outcomes and authors’ conclusions are presented in Table 5.

**Table 4:** ISS to improve clinical skills and outcomes

Author, date (Country)	Research topic	Setting and participants	Outcome methods and measures	Study type *Quality assessment* (JBI level of evidence)
**Andreatta 2011 **[87]** (US)**	Viability and effectiveness of a simulation-based paediatric mock code program on patient outcomes, as well as residents’ confidence in performing resuscitations	Children’s hospital (*n* = 41)	1. Survival rates	Prospective observational *Quality assessment – moderate* (3e)
**Barni 2018 **[79]** (Italy)**	Improve management of anaphylaxis	Paediatric ED (*n* = 30)	1. Clinical management	Prospective observational *Quality assessment—good* (3e)
**Ben-Ari 2018 **[80]** (Israel)**	Improve safety practice of ED sedation	ED (*n* = 16)	1. Sedation performance scores	Prospective observational *Quality assessment: moderate* (3e)
**Braddock 2014 **[90]** (US)**	Improve clinical outcomes and safety culture	Inpatient units (*n* = 303)	1. Incidence of septic shock 2. Incidence of respiratory failure	Prospective observational *Quality assessment: good* (3e)
**Coggins 2019 **[86]** (Australia)**	Improve mechanical CPR performance	(*n* = 112)	1. CPR performance scores	RCT *Risk of bias: moderate* (1c)
**Generoso 2016 **[91]** (US)**	Improve nurses' responses in the first 5 min of in-hospital emergencies	(*n* = 147)	1. Clinical management	Prospective observational *Quality assessment: moderate* (3e)
**Gibbs 2018 **[88]** (US)**	Diagnose and correct LST to mitigate a methicillin-resistant Staphylococcus aureus outbreak	NICU (*n* = 99)	1. Hand hygiene 2. MRSA outbreaks	Prospective observational *Quality assessment: good* (3e)
**Hamilton 2015 **[93]** (US)**	Improving technical and interprofessional skills during an emergent simulated open thoracotomy		1. Time taken to complete procedure	Prospective observational *Quality assessment: moderate* (3e)
**Josey 2018 **[85]** (US)**	Survival rates following In-hospital cardiac arrest for hospitals more and less active in in-situ mock code training	26 Hospital sites	1. Survival rates	Prospective observational *Quality assessment: good* (3e)
**Knight 2014 **[84]** (UK)**	Improving survival to discharge and code team performance after paediatric in-hospital cardiopulmonary arrest	(*n* = 169 patients. CG = 123 / IG = 46)	1. Survival 2. Neurological morbidity 3. Adherence to standards	Observational with historical controls *Quality assessment: moderate* (3e)
**Kobayashi 2012 **[81]** (US)**	Determine baseline performance of ED telemetry for detecting arrhythmias and improve system performance through human factors engineering (HFE)	ED	1. Detection of ventricular tachycardia and sinus bradycardia	Prospective observational *Quality assessment: moderate—good* (3e)
**Lavelle 2017 **[2]** (UK)**	To improve management of medical deterioration	mental health settings (*N* = 53)	1. Incident rates	Prospective observational *Quality assessment: moderate* (3e)
**Marshall 2015 **[94]** (US)**	To improve team training for postpartum haemorrhage	Community maternity hospitals (*n* = 22)	1. Clinical management 2. Response times	Prospective observational *Quality assessment: moderate* (3e)
**Sleeman 2018 **[92]** (UK)**	To improve the identification and treatment of hypoglycaemia	Hospital ward	1. Number of incidents	Prospective observational (QI) Quality *assessment: low—moderate* (4)
**Steinemann 2011 **[82]** (US)**	Evaluate the impact of a team training curriculum on team communication, coordination and clinical efficacy of trauma resuscitation	ED (*n* = 137)	1. Resuscitation time	Prospective cohort *Quality assessment: moderate* – good (3e)
**Sullivan 2015 **[83]** (US)**	Improve retention of cardiopulmonary resuscitation priorities for in-hospital cardiac arrests	(*n* = 72)	1. Clinical management 2. Response times	RCT *Risk of bias: moderate* (Ic)
**Theilen 2013 **[89]** (UK)**	Evaluate the long-term impact of ongoing regular team training on hospital response to deteriorating ward patients, patient outcome and financial implications	PICU (admissions *n* = 139)	1. Response times 2. Clinical management 3. Transfer times	Prospective cohort *Quality assessment: moderate* (3e)

*ED* Emergency Department, *ICU* Intensive Care Un it, *MDT* Multi Disciplinary Team, *PICU* Paediatric Intensive Care Unit, *OR* Operating Room, *CICU* Cardiac Intensive Care Unit

**Table 5:** ISS to improve clinical skills and patient outcomes: ISS Frequency and authors conclusions

ISS approach	Author year	ISS focus	Number of ISS (length)	Summary of authors conclusions
**Clinical skills**	Coggins 2019 [86]	Mechanical CPR	3 sessions with 4 month follow-up in the CG	Providers receiving additional simulation-based training had higher retention levels of M-CPR skills
	Sullivan 2015 [83]	CPR	Every 2, 3 and 6 months	Short ISS training sessions conducted every 3 months are effective in improving timely initiation of chest compressions and defibrillation
	Ben-Ari 2018 [80]	Sedation procedures	1 session	Sedation simulation training improves several tasks related to patient safety during sedation
	Hamilton 2015 [93]	Open thoracotomy	3 sessions	ISS appears useful for improving team performance during simulated bedside OT
**Clinical management Response times**	Steinemann 2011 [82]	Trauma resuscitation	1 × 3 h (30-min scenario + 150 min debrief)	A relatively brief simulation-based curriculum can improve the teamwork and clinical performance of trauma teams
	Theilan 2013 [89]	Response to deteriorating patients	Weekly (attendance at 10 per year)	Lessons learnt during team training led to sustained improvements in the hospital response to critically deteriorating in-patients, significantly improved patient outcomes and substantial savings
	Barni 2018 [79]	Management of anaphylaxis	4 sessions over 3 months (1 h)	ISS improved the correct management of anaphylaxis and led to a higher number of patients being referred to the allergy unit for evaluation
	Sleeman 2018 [92]	Hypoglycaemia Identification and treatment	Not reported	Hypoglycaemia ISS training is a positive addition in the education of healthcare professionals. ISS intervention demonstrated favourable outcomes
	Generoso 2016 [91]	Nurses’ response to emergencies	1 session (30 min)	Establishing ISS is feasible and well received. This approach appears effective in increasing confidence, initiating life-saving measures, and empowering nurses to manage emergencies
	Marshall 2015 [94]	Management of Postpartum Haemorrhage	1 ISS repeated at 9–12 months	Simulation and team training significantly improved postpartum haemorrhage response times among clinically experienced community labour and delivery teams
**Survival rates, incidents and outcomes**	Knight 2014 [84]	Responding to paediatric cardiac arrest	16 sessions over 18 months	With implementation of Composite Resuscitation Team Training, survival to discharge after paediatric cardiopulmonary arrest improved, as did code team performance
	Braddock 2014 [90]	Safety culture and outcomes	4 sessions per month	A multifaceted patient safety program suggested an association with improved hospital acquired complications and weighted, risk-adjusted mortality, and improved nurses’ perceptions of safety culture on inpatient study units
	Andreatta 2011 [87]	Resuscitation outcomes	Monthly over 48 months	Simulation-based mock code program may significantly benefit paediatric patient outcomes
	Gibbs 2018 [88]	Mitigate a MRSA outbreak	1 session (30 min)	ISS can counter threats to patient safety related to workflow and lapses in infection control practices and improve patient outcomes
	Lavelle 2017 [2]	Manage deteriorating patient	Weekly sessions	ISS for medical deterioration yielded promising outcomes for individuals and teams
	Josey 2018 [85]	Cardiac arrest survival	Not reported	Hospitals with more active ISS participation had higher survival rates than hospitals with less-active ISS participation although the findings should be considered with caution due to the limitations in collecting hospital level data and potential bias from other confounding factors
Use of equipment	Kobayashi [81]	Telemetry for detecting arrhythmias	50 sessions over × 3 2-week periods	Experimental investigations helped reveal and mitigate weaknesses in an ED clinical telemetry system implementation

Some studies which involved more complex practices and clinical outcomes implemented regular ISS interventions over longer time periods. Andreatta et al. [87] conducted paediatric mock codes (resuscitation scenarios), on a monthly basis for 48 months and reported hospital survival rates improved significantly over study period. Knight et al. [84] conducted 16 paediatric ISS sessions over 18 months and reported that survival rates had improved when compared to historical controls. Other studies reporting favourable outcomes for regular ISS training included anaphylaxis management [79], sepsis management [90] response times to hospital emergencies [91], detection of arrhythmias [81], management of medical deterioration [2, 89] and CPR performance [83, 86].

Studies which included more easily defined or isolated tasks, reported one to three ISS sessions as effective in improving: infection control practices [26]; thoracotomy procedures [93]; response times and management of PPH [94]; sedation practices [80]; and resuscitation response times [82].

### ISS to improve non-technical skills, knowledge and comfort and confidence

Non-technical Skills (NTS) are individual and team social and cognitive skills, hat support technical skills when performing complex tasks. NTS can include planning and preparation for complex tasks, situation awareness, perception of risk, decision-making, communication, teamwork and leadership [95]. Twenty-seven studies reported ISS interventions to improve NTS, participant comfort and confidence (Table 6). Studies were conducted in Australia, Canada, Denmark, France, the UK and US. Sixteen studies were observational; there was one prospective cohort study, five RCTs, and five quasi-experimental studies. Studies were conducted in adult and paediatric emergency and resuscitation teams and departments [69, 71, 82, 96–105], paediatric and neonatal care [106–112], obstetric care [24, 113–115], ICU [116, 117], a post anaesthesia care unit [118] and a mental healthcare setting [2]. Where reported, ISS interventions were delivered over periods of one day to 18 months, with training lasting from 30 min to 3 h. Reported sample sizes ranged from 20—750 participants.

**Table 6:** ISS to improve non-technical skills, knowledge and comfort and confidence

Author, date (Country)	Research topic	Setting and participants	Outcome methods and measures	Study type (JBI level of evidence)
**Allan 2010 **[111] (US)	Improving caregiver comfort and confidence levels regarding future resuscitation events	ICU (*n* = 182)	1. Function as a team member / leader 2. Confidence 3. Anxiety 4. Preparedness to alert team leader	Prospective observational *Quality assessment – moderate – good* (3e)
**Bayouth 2018 **[106] (US)	To identify targets for educational intervention and increase provider experience of paediatric trauma simulations		1. Comfort scores 2. Performance scores	Prospective observational *Quality assessment – moderate – good* (3e)
**Boyde 2018 **[104] (Australia)	To implement and evaluate an innovative simulation experience for registered nurses	(*n* = 50)	1. Anxiety 2. Clinical performance	Prospective observational *Quality assessment: moderate* (3e)
**Cepeda 2017 **[97] (US)	Improve provider proficiency and confidence in the performance of neonatal resuscitation with a focus on chest compression effectiveness	(*n* = 25)	1. Confidence scores 2. Proficiency scores	Quasi-experimental *Quality assessment – moderate* (2d)
**Crofts 2007 **[115] (UK)	To explore the effect of obstetric emergency training on knowledge. To assess if acquisition of knowledge is influenced by the training setting or teamwork training	Maternity unit (*n* = 140)	1. Knowledge scores	RCT *Risk of bias: moderate* (1c)
**Davison 2017 **[98] (Australia)	Orientate staff prior to opening a new paediatric emergency service	ED (*n* = 89)	1. Confidence scores 2. Orientation scores	Prospective observational *Quality assessment – moderate (*3e)
**Dowson 2013 **[112] (UK)	Improve the management of paediatric emergencies improves qualified nurses’ clinical confidence	(*n* = 20)	1. Technical scores 2. Non-technical scores 3. Management scores 4. Confidence scores	Quasi-experimental *Quality assessment – good* (2d)
**Freund 2019 **[105] (Denmark)	Perception of learning and stress comparing announced and unannounced ISS	ED / Trauma (*n* = 130)	1. Learning scores 2. Stress scores 3. Unpleasantness scores 4. Anxiety scores	Quasi-experimental *Quality assessment – moderate—good* (2d)
**Author, date (Country)**	**Research topic**	**Setting and participants**	**Outcome methods and measures**	
**Gardner 2013 **[71] (US)	To identify if ISS can impact important employee perceptions and attitudes in a new facility	ED (*n* = 55)	1. Communication scores 2. Self-efficacy 3. Performance beliefs	Prospective observational *Quality assessment – good* (3e)
**Gundrosen 2014 **[116] (Norway)	To assess the feasibility of ISS and assessing non-technical skills	ICU (*n* = 72)	1. Knowledge and confidence scores 2. Compliance with the WHO Surgical Safety Checklist	Feasibility RCT *Risk of bias: moderate* (1c)
**Katznelson 2014 **[99] (US)	ISS to increase provider comfort with seriously ill children	ED (*n* = 69)	1. Comfort scores	Prospective observational *Quality assessment – moderate (*3e)
**Katznelson 2018 **[100] (US)	In-situ paediatric simulation in on care team performance during resuscitation scenarios	Hospital (hospital *n* = 5)	1. Performance scores	Prospective observational *Quality assessment – good (*3e)
**Kurosawa 2014 **[108] (US)	Feasibility and effectiveness of ISS Paediatric Advanced Life Support training for recertification	Paediatric (*n* = 40)	1. Clinical performance scores 2. Behavioural scores	RCT *Risk of bias: moderate* (1c)
**Lavelle 2017 **[2] (UK)	To improve management of medical deterioration in mental health settings	Mental Health Settings (*n* = 53)	1. Knowledge 2. Confidence 3. Attitudes	Prospective observational *Quality assessment: moderate* (3e)
**Nickerson 2019 **[113] (US)	Improve knowledge, confidence, and clinical skills in performing manoeuvres to reduce a shoulder dystocia and neonatal resuscitation	ED (*n* = 52)	1. Knowledge 2. Confidence 3. Clinical skills	Prospective observational *Quality assessment: moderate* (3e)
**Nunnink 2009 **[117] (Australia)	Evaluate the impact on knowledge and confidence of team-based chest reopen training using a patient simulator	ICU (*n* = 49)	1. Knowledge scores 2. Confidence scores	Quasi-experimental *Quality assessment: good* (2d)
**Patterson 2013 **[69] (US)	To decrease the frequency and mitigate the effects of medical error	Paediatric ED (*n* = 289 / *n* = 151)	1. Knowledge scores	Prospective observational *Quality assessment: good* (3e)
**Patterson 2013b **[101] (US)	Promote identification of LSTs and systems issues at a higher rate than seen in the simulation lab setting	ED (*n* = 218)	1. Perceived value 2. perceived impact 3. Non-technical skills	Prospective observational *Quality assessment: moderate—good* (3e)
**Rubio-Gurung 2014 **[24] (France)	Improve neonatal resuscitation performed by the staff at maternities	Maternity unit (*n* = 49)	1. Technical scores 2. Performance scores	RCT *Risk of bias: moderate* (1c)
**Author, date (Country)**	**Research topic**	**Setting and participants**	**Outcome methods and measures**	
**Saqe-Rockoff 2019 **[96] (US)	Improve nurse’s competence and self-efficacy in paediatric resuscitation scenarios using a low-fidelity simulation	(*n* = 43)	1. Confidence scores 2. Performance scores	Prospective observational *Quality assessment: good* (3e)
**Siegel 2015 **[102] (US)	Investigation of Emergency Department Procedural Sedation (EDPS) testing an informatics system	ED (*n* = 24)	1. Situational awareness scores	RCT *Risk of bias: low—moderate* (1c)
**Steinemann 2011 **[82] (US)	Evaluate the impact of a team training curriculum on team communication, coordination and clinical efficacy of trauma resuscitation	ED (*n* = 137)	1. NONTECHS (non-technical skills) scores	Prospective cohort *Quality assessment: moderate—good* (3e)
**Stocker 2012 **[109] (UK)	To evaluate the impact of ISS on perceived performance	PICU (*n* = 219)	1. Perceived impact 2. Non-technical skills scores 3. Technical skills 4. Confidence	Prospective observational *Quality assessment: moderate—good* (3e)
**Surcouf 2013 **[114] (US)	Improve residents’ self-confidence and observed performance of adopted best practices in neonatal resuscitation	(*n* = 27)	1. Self-confidence scores 2. Performance scores	Prospective cohort *Quality assessment: moderate—good* (3e)
**Van Schaik 2011 **[103] (US)	Interprofessional team training in Paediatric resuscitation to enhance self-efficacy among participants	Paediatric	1. Confidence scores	Prospective observational *Quality assessment: moderate—good* (3e)
**Villemure 2019 **[118] (Canada)	ISS training on interprofessional collaboration during crisis event management in post-anaesthesia care	Post anaesthesia care unit	1. Collaboration scores 2. Communication scores	Quasi-experimental *Quality assessment: good* (2d)
**Von Arx 2010 **[110] (US)	Improving participant comfort and subjective knowledge of paediatric codes	(*n* = 27)	1. Comfort scores 2. Knowledge scores	Prospective observational *Quality assessment: moderate* (3e)

*ED* Emergency Department, *ICU* Intensive Care Unit, *MDT* Multi Disciplinary Team, *PICU* Paediatric Intensive Care Unit, *OR* Operating Room, *CICU* Cardiac Intensive Care Unit

Outcome measures included self-reported confidence scores, performance scores, management and leadership scores, communication, and self-reported anxiety and knowledge. Outcome measures, ISS frequency and outcomes scores are presented in Table 7.

- Significant improvements in confidence scores were reported for single session [96, 98, 111, 114], three session [112, 117] or regular departmental training [2].
- Improvements in participants’ performance scores were reported in six studies [24, 71, 96, 104, 108, 113], with most studies conducting a single ISS intervention.
- Two studies reported significant improvements in participants management and leadership scores following a single session [111] and three session ISS intervention [112].
- Two studies [71, 118] reported an improvement in communication scores following 1–3 ISS interventions.
- Two studies reported significant improvement in anxiety scores following a single ISS intervention [104, 111].
- Four studies reported a significant improvement in participants knowledge scores following a brief ISS intervention [2, 101, 113, 115].

**Table 7:** Confidence, performance, management, communication, anxiety and knowledge scores reported in the included studies

Confidence scores			
**Davison 2017 **[98] 1 × ISS (*n* = 89) *Study specific questionnaire*	Pre Mean Scores (SD)	Post Mean Scores (SD)	Significance
	28.8 (6.3)	30.8 (4.6)	< 0.001
**Allan 2010 **[111] 1 × ISS (*n* = 182) *Study specific questionnaire*	Pre v Post		< 0.001
**Lavelle 2017 **[2] Regular weekly ISS (*n* = 53) *Study specific questionnaire*	3.6 (0.9)	4.1 (0.9)	< 0.001
**Nickerson 2019 **[113] 1 × 15 min ISS (*n* = 23) *Study specific questionnaire*	1.4	2.8	NR
**Saqe-Rockoff 2019 **[96] 1 × ISS (*n* = 43) *C-Scale (Grundy 1993)*	2.5 (0.8)	3.9 (0.6)	< 0.001
**Surcouf 2013 **[114] 1 × ISS (*n* = 27) *Study specific questionnaire*	2.53 (0.46)	2.92 (0.56)	< 0.001
**Van Schaik 2011 **[103] Regular interprofessional team training (monthly – quarterly) *Study specific questionnaire*	Basic PGY 1/2/3 3.59 (0.56) 4.08 (0.44) 4.12 (0.5) Advanced PGY1/2/3 2.35 (0.6) 2.81 (0.6) 2.71 (0.52) Expert PGY1/2/3 1.5 (0.76) 1.73 (0.82) 1.44 (0.57) Leadership PGY1/2/3 1.88 (0.79) 2.77 (0.62) 3.06 (0.91)	Basic PGY 1/2/3 3.73 (0.6) 3.97 (0.44) 4.36 (0.37) Advanced PGY1/2/3 2.52 (0.67) 2.68 (0.6) 3.17 (0.51) Expert PGY1/2/3 1.75 (0.71) 1.54 (0.8) 1.95 (0.84) Leadership PGY1/2/3 2.32 (0.88) 2.84 (0.61) 3.57 (0.62)	0.301 0.110 0.156 0.011
**Dowson 2013 **[112] IG 3 × ISS (*n* = 20) *Clinical Confidence Rating Scale *[108]	Month 1 CG 57.8 (10.7) Month 1 IG 47.3 (6.68)	Month 3 CG 60 (10) Month 3 IG 56.6 (7)	NS < 0.001
**Nunnink 2009 **[117] 3 × ISS and 2 × video training (*n* = 49) *Study specific questionnaire*	ISS 9 (4.3) Video 11.2 (3.8)	ISS 12.9 (3.6) Video 12.4 (4.1)	0.001 0.03
**Performance scores**			
**Gardner 2013 **[71] 1 × ISS *Study specific questionnaire*	3.72 (0.53)	3.52 (0.7)	< 0.001
**Nickerson 2019 **[113] 1 × ISS 15 min (*n* = 23) *Study specific questionnaire*	67% 62%	86% 89%	Not reported
**Saqe-Rockoff 2019 **[96] 1 × ISS (*n* = 43) *Clinical Performance Tool (Donoghue *et al*. 2010)*	5.3 (0.9)	9.2 (0.6)	0.004
**Boyde 2018 **[104] 1 × ISS (*n* = 50) *Self-Efficacy in Clinical Performance scale (Munroe *et al*., 2015)*	165.15 (28.1)	214.12 (26)	< 0.001
**Rubio-Gurung 2014 **[24] IG 1 × 4-h ISS training session (*n* = 120) *Team Emergency Assessment Measure (TEAM) (Cooper *et al*. 2010)*	CG post Median 6.7 (3.4–8.3)	IG post Median 19.9 (13.3–25)	0.001
**Kurosawa 2014 **[108] 6 × ISS (*n* = 40) *Validated Clinical Performance Tool*	CG post 14.9 (4.4)	IG post 22.4 (3.9)	0.001
**Management and leadership scores**			
**Allan 2010 **[111] 1 × ISS (one component of improvement project) (*n* = 182) *Study specific questionnaire*	NR	NR	< 0.001
**Dowson 2013 **[112] IG 3 × ISS training (*n* = 20) *Clinical Confidence Rating Scale*	Month 1 CG 2.9 (0.57) Month 1 IG 2.2 (0.42)	Month 3 CG 2 (0.7) Month 3 IG 2.8 (0.4)	NS < 0.05
**Communication scores**			
**Gardner 2013 **[71] at least1 x ISS Q*uestionnaire developed by O’Neill *et al*. (1994)*	3.64 (0.64)	3.82 (0.6)	< 0.05
**Villemure 2019 **[118] IG 6 h training: including 3 × ISS scenarios 30 min each *Work Collaborative Questionnaire (Chiocchio *et al*. 2012)*		CG 5.27 (0.95) IG 4.9 (0.91)	NS
**Anxiety scores**			
**Allan 2010 **[111] 1 × ISS as one component of improvement project (*n* = 182) *Study specific questionnaire*	Pre / Post		< 0.001
**Boyde 2018 **[104] 1 × ISS (*n* = 50) *State-Trait Anxiety Inventory (STAI) Anxiety State (Spielberger *et al*. 1983)*	38.56 (9.87)	33.54 (9)	< 0.001
**Knowledge scores**			
**Crofts 2007 **[115] Single ISS (comparing in situ *n* = 64, with simulation centre training *n* = 69, without and without teamwork training) *Study specific questionnaire*	In situ pre 81.5 (21.3) Simulation centre pre 79.4 (22.1)	In situ post 101.5 (21.5) Simulation centre post 100.5 (21.1)	NS difference between ISS and sim centre
**Lavelle 2017 **[2] Regular weekly ISS (*n* = 53) *Study specific questionnaire*	38.6 (19.3)	53 (16)	< 0.001
**Nickerson 2019 **[113] 1 × 15 min ISS *Study specific questionnaire*	57%	72%	Not reported
**Patterson 2013b **[101] 2 day education intervention with ISS (*n* = 289), re-evaluation at 10 months (n-151) *Study specific questionnaire*	86% (SD 9.8%)	96% (SD 5.8%) Re-evaluation 93% (SD 7.3%)	< 0.001

*NR* Not Reported, *NS* Not Significant, *IG* Intervention Group, *CG* Control Group, *SD* Standard Deviation

Rubio-Gurung et al. [24] compared a four-hour ISS intervention to improve neonatal resuscitation across maternity units with control groups (*n* = 12, 6 units in each group). The median technical score was significantly higher for the ISS groups compared to the control groups. In the ISS groups, the frequency of achieving a heart rate of 90 per minute at 3 min improved significantly and the number of hazardous events decreased significantly. Four studies which compared ISS groups with control or comparison groups reported no statistical significant difference in outcomes: Gundrosen et al. [28] compared nurses one hour lecture-based training with ISS training on participants situational awareness and team working (ANTS taxonomy); Crofts et al. [115] compared a ISS intervention for obstetric emergency management with training conducted in a simulation centre; Villemure et al. [118] compared ISS in post anaesthetic care units with a control group (no particular interprofessional education).; Dowson et al. [112] compared regular ISS training to improve nurses’ clinical confidence in the management of paediatric emergencies with a control group (mandatory resuscitation training).

### ISS settings and methods

Studies conducted ISS interventions in in-patient care settings, predominantly in adult and paediatric EDs, obstetric/maternity units, cardiac response teams, adult and paediatric ICUs, and operating rooms. Data collection methods included direct observation, video review and data collected from simulation or clinical equipment. Participants’ knowledge, anxiety, comfort and safety attitudes were exclusively measured by self-reported questionnaires. There was a range of methods between and within studies to measure task performance, clinical management, teamwork and communication (including assessment from direct or video observation), alongside participants’ self-reported outcomes and /or clinical outcomes data.

Studies used various tools to assess performance during ISS interventions including:

- Teamwork and non-technical skills: Simulation Team Assessment Tool STAT [65], NONTECHS [119], Anaesthetists' non-technical skills (ANTS) taxonomy and behaviour rating tool [67], TeamSTEPPS [68], TeamMonitor [120], Clinical Teamwork Scale [121], Team Emergency Assessment Measure (TEAM) [122]
- Readiness scores: TESTPILOT [78], Emergency Medical Services for Children Readiness Survey [123]
- Clinical performance: Clinical performance during Paediatric Advanced Life Support simulation scenarios [124], Self-Efficacy in Clinical Performance scale [125]
- Confidence scale [126]
- Communication and collaboration [127]

The benefits and limitations of conducting ISS reported across all included studies are summarised in Table 8.

**Table 8:** Benefits and limitations of ISS reported in the included studies

Benefits	Limitations
Realism: Real setting enabling teams to perform with actual equipment and resources Locate and test equipment Facilitates safe transitions to new facilities	Possibility of selection bias / lack of randomisation of participants
	Releasing participants from other clinical duties while undertaking ISS may limit generalisability to the clinical setting
	Possibility of training effect for pre-announced ISS: enabling participants to prepare (as opposed to unannounced ISS)
	Observers and video reviewers are unblinded to the type of participant and setting
	Lack of usual clinical distractions and lack of assessment over the full 24-h period may limit generalisability
	High cancellation rate in high acuity areas
	Fidelity issues in key components of task completion (lack of adequate visual cues regarding patient output, monitor function and appearance)
Scenarios can be rated independently my numerous assessors	Small sample sizes and inadequately powered studies prevents formal statistical analysis
	Problems with recruitment
	Use of non-validated assessment tools
	Confounding factors: unable to capture all of the complex all factors which contribute to outcomes in a changing climate of practice
Some tasks are capable of high fidelity and reproducibility	Inadequate collection of participant demographic data which may impact the findings (e.g., number of shifts worked or days off before the data collection, participation in more than one scenario, prior simulation training)
Assessment of tasks with clearly defined and established standards	Potential ‘refresher effect’ if participants repeatedly engage in ISS simulations
	Efforts to standardise ISS activities may limit including variation between scenarios and tasks
	Evaluation of ISS assessment in one setting reduces generalisability to the wider context
Identified opportunities for improvement in the clinical setting	Lack of formal measures to translate the findings into practice and inform action plans
Enables more team members to participate compared to off-site training	Variation in teams when evaluating pre / post assessments over longer follow-up periods
	Measuring communication in an established team maybe difficult as the need for communication decreases
	Lack of availability of experienced non-technical skills assessors
	Maintaining participant anonymity in smaller sites / studies
	Performance anxiety, reluctance to participate

## Discussion

This systematic mapping review found that ISS is reported to be feasible and beneficial in a variety of inpatient clinical settings. It is used to assess a number of different domains of practice including adherence to clinical guidelines and standards, task completion times, team performance, non-technical skills, detection of errors and latent safety threats.

Lamé and Dixon-Woods [128] make an important distinction between research which is conducted about simulation and research conducted through simulation. The findings from this review include both of these approaches, which at times overlap, studied though various experimental designs. Research conducted about ISS (where ISS was an active intervention) included studies exploring acceptability and usefulness of ISS to clinicians and educators and evaluating the ability of ISS to identify LSTs and improve individual, team and system-level outcomes. Research conducted through ISS often included ISS as part of a multicomponent approach to improve clinical skills, performance and outcomes.

ISS outcomes were used to highlight where additional or new methods of training might be required to improve the quality of care, to identify LSTs and explore the accuracy and efficiency of task completion over the period of a working shift. Exploring the factors that can affect variations in adherence to clinical procedures, outcomes and performance may help to uncover where and why errors occur. ISS has the potential to reveal the constraining and facilitating mechanisms which impact performance and to identify modifiable factors at the individual, departmental, institutional level or system level [52–54].

Some multicentre studies were conducted to assess clinical performance used validated tools to assessed adherence to guidelines and departmental readiness scores. The ability to standardise simulation across participating sites can help isolate independent variables and to reduce the risk of bias introduced by variations in local contexts [129]. Differences in performance can be explored between sites and be used to generate theory about why differences may occur. For example, Auerbach et al. [53] used ISS to explore hospital characteristics to adherence to paediatric cardiac arrest guidelines across four paediatric EDs. ISS outcomes based on clinical standards can serve as a proxy for real performance, enhancing the external validity of the study findings [54].

There were considerable variations in the frequency of ISS sessions, length of ISS sessions and use of announced and unannounced ISS. However the length and frequency of ISS were not always reported. Studies which are focused on relatively straightforward, easily defined or isolated tasks, see improved outcomes after one to three ISS sessions [80, 82, 88, 93, 94]. Studies involving more complex practices or outcomes seem to require interventions over longer time periods [2, 79, 84, 87]. This may indicate a potential benefit of ISS to support complex skills acquisition through behavioural learning strategies, where skills are developed through repetition and behaviour change occurs through feedback from the simulation activity, interaction between the task, environment, and the team.

Most of the studies included in the review used locally developed checklists, developed through previous pilot testing or amended from checklists developed for other clinical settings. In general, there was a paucity of reporting of the validity and reliability of assessment measures and tools. Studies which reported team and system level assessments adopted more established outcome measures [65, 67, 68, 120, 121, 123]. Measurement methods for assessing individual competencies involved in complex care processes are less well-defined, and further complicated when individual performance needs to be isolated from the wider team. Concepts such as ‘effective communication’ are subject to interpretation and clinical outcomes may be attributed to concepts such as teamwork, communication and leadership in addition to clinical skills and knowledge [30]. Griswold et al. [29] identify that for clinical procedures with clear chains of action and well-defined processes and standards, summative ISS assessment is much simpler than in more “dynamic, multifactorial practices in which cognitive, procedural, and communication skills are simultaneously applied in a team environment” (Griswold et al. 2017, page 170). Criterion standards and benchmarks of quality performance need to be further developed to reliably and accurately capture the individual performance which is linked to relevant clinical competencies.

Goldstein et al. [130] stated that literature reporting ISS interventions on patient outcomes is scarce. Surrogate endpoints, such as response times are frequently adopted but this does not truly represent the complex factors that lead to improved patient outcomes [130]. In this review, ISS was often incorporated within larger, multi-component educational improvement projects. Most studies were observational with only thirteen adopting experimental designs. Small, observational studies are often limited by the potential for introducing selection bias, observer bias and confounding. Lamé & Dixon-Woods [128] state that ISS which can reproduce situations identically before and after the intervention increases confidence that the intervention can explain the variation in outcomes. Time-series designs which collect data at multiple times before and after the intervention or controlled studies are required to provide greater confidence in the findings of ISS interventions [128].

Unannounced ISS (or mock drills) were mainly conducted where studies sought to carry out a system audit or to assess clinical performance against a benchmark. Whereas announced ISS, which gave participants varying levels of notice and access to supportive resources, were mainly conducted as part of improvement projects or as part of clinical training. Posner et al. [32] highlight that both announced and unannounced ISS approaches can be conducted to detect LSTs, although assessment of factors such as response times and leadership assignment are more suited to unannounced ISS [55, 58]. Freund et al. [105] compared unannounced to announced (one hour prior to ISS) team training and reported no significant differences on self-perceived learning and self-reported stress outcomes. It is reported that ISS can pose numerous threats an individual’s psychological safety which can have a negative effect on learning. Participants may feel under increased scrutiny from colleagues or burdened by their other clinical work. Psychological safety can be supported by including a pre-simulation brief to discuss training objectives, expectations and develop trust between educators and learners [32, 131, 132].

Cheng et al. [129] recommend an extension to the CONSORT guidelines for reporting simulation-based research to include demographics and clinical characteristics of participants and the setting. This should include participants’ previous experience with simulation, skill mix, staffing, capacity pressures and other relevant features to facilitate an assessment of the external validity of the findings [53]. A review by Goldshtein et al. [130] reported that it was difficult to assess who was participating in ISS and their prior experience of ISS participation. Lipman et al. [53] reported that clinical timings evaluated in their study may have been underestimated due to participation of highly skilled teams, the close proximity of clinical departments and participants to the drill area, absence of patient family members, participant knowledge of the imminent ISS activity and the daytime hours [55]. In future studies, detailed information on other potential sources of bias and other confounding, contextual and system level factors should be presented to assist researchers, educators and clinicians to assess the relevance of the findings to other settings and participant groups [129].

ISS to assist teams train, rehearse and practice for low frequency, high impact events were frequently reported simulation activities in the review. The theoretical base for ISS as a training intervention was not reported in many studies, however ISS as a training intervention maps to the concepts within cognitive learning approaches where participants preconceptions are explored, and new or unexpected events are presented via the simulation activity to challenge precognitions [133]. ISS is also underpinned by situativity theory, in which knowledge transfer is considered optimal when the learning environment matches the environment in which it will be applied [28, 131, 134]. During the Covid-19 pandemic, ISS has been used to help staff prepare for emerging challenges. ISS interventions have helped to identify LSTs, highlight inadequacies in guidelines and protocols policies, improve the correct use of PPE, and orientate staff to newly established Covid-19 intensive care unit and wards [135, 136].

### Study strengths and limitations

This review should be viewed in light of several limitations. This review did not include grey literature, conference abstracts and academic theses. It is likely that grey literature may include ISS practice-based improvement and educational projects which further illustrate the current uses of ISS in healthcare settings. However, this review highlights the lack of rigorous intervention ISS research and the urgent need to increase research output and methodological quality. The mapping review aimed to provide an overview of the broad ISS published literature and did not conduct in-depth analysis of study outcomes to enable meaningful comparisons. The review has highlighted different categories and approaches to ISS, identifying common outcomes measures and measurement tools. Mapping reviews are distinguished by the presentation of the data in a digestible format and assessment of whether the total population of studies is similar enough to undertake a coherent synthesis of the current data [36]. Therefore, this review may provide a useful starting point for other researchers seeking to develop and define parameters for future ISS systematic reviews.

## Conclusion

This review presents an overview of the literature on ISS interventions by mapping the study objectives, methods, outcomes, barriers, and facilitators at work across different settings. The mapping review provides a useful summary for healthcare educators and researchers seeking to develop ISS strategies in healthcare settings. Additionally, it highlights important evidence gaps, including the need to (1) identify appropriate tasks capable of standardisation and reproducibility in ISS assessment scenarios (2) capture adequate demographic data from participants to assess the impact on outcomes (e.g. work-patterns, skill-mix, experience, ISS experience and exposure, willingness to participate) (3) explore different methodologies in an attempt to reduce bias and confounding factors (4) develop and validate sensitive data collection methods and tools to capture the complexity of team and individual performance in real settings (5) identify optimal frequency and length of time to complete ISS, considering feasibility and acceptability in the clinical setting. This systematic mapping review has provided a useful framework to navigate the expansive and diverse research literature on a relatively new and underdefined approach to ISS as a function to assess individual, team and departmental performance. There is currently a lack of consensus for the rationale for conducting ISS interventions and well-developed studies are required to identify the potential benefits of ISS and the impacts on patient outcomes. Overall, studies reported ISS to be feasible and beneficial to address various learning and improvement objectives. The components and mechanisms employed across the included studies which have been designed to address a range of objectives can inform future design of ISS interventions to meet specific objectives.

## Data Availability

All data generated or analysed during this study are included in this published article [and its supplementary information files].

## Abbreviations

ISS: In-situ simulation
MDT: Multidisciplinary team
LST: Latent safety threat
PPE: Personal protective equipment
ED: Emergency Departments
ICU: Intensive Care Unit
CPR: Cardiopulmonary resuscitation
